# Clinical and Radiological Outcomes of Operative Therapy in Insertional Achilles Tendinopathy With Debridement and Double-Row Refixation

**DOI:** 10.1177/10711007211002814

**Published:** 2021-04-10

**Authors:** Fabian Greiner, Hans-Jörg Trnka, Michel Chraim, Elena Neunteufel, Peter Bock

**Affiliations:** 1 Department of Orthopaedics and Trauma-Surgery, Medical University of Vienna, Vienna, Austria; 2 Department of Paediatric Orthopaedics, Adult Foot and Ankle Surgery, Speising Orthopaedic Hospital, Vienna, Austria; 3 Fusszentrum Vienna, Vienna, Austria; 4 Orthopoint Vienna, Vienna, Austria

**Keywords:** Achilles tendinopathy, insertional, Achilles tendon

## Abstract

**Background:**

Insertional Achilles tendinopathy (IAT) is a painful pathology in which the strongest and thickest tendon of the human body is affected. Different conservative and operative treatments have been described to address this pathology. This study aimed to evaluate the medium-term clinical and radiological outcomes of patients who underwent a surgical therapy via a longitudinal tendon-splitting approach with debridement and double-row refixation.

**Methods:**

All patients were assessed pre- and postoperatively using a visual analog scale (VAS), the American Orthopaedic Foot & Ankle Society (AOFAS) Hindfoot Score, the Foot and Ankle Outcome Score (FAOS), and the Foot Function Index (FFI). Additionally, a lateral radiograph of the foot was performed to assess the postoperative result. Forty-two patients with confirmed IAT who underwent surgery between 2013 and 2017 with a longitudinal tendon-splitting approach and tendon refixation using a double-row refixation system were evaluated. The average follow-up was 32.8 (range, 18-52) months. We included 26 female and 16 male patients with an average age of 56.8 (range, 27-73) years.

**Results:**

The mean VAS improved from 8.91 ± 1.0 preoperatively to 1.47 ± 2.5 postoperatively (*P* **<** .01). AOFAS scores improved significantly from 51.0 ± 12.5 preoperatively to 91.3 ± 14.3 postoperatively (*P* **<** .01). All total and subscores of the FFI and FAOS saw a significant improvement at follow-up (*P* **<** .01). Lateral radiographs showed recurrent calcification in 30 patients (71.4%).

**Conclusion:**

We found that, at an average of 33 months posttreatment, insertional Achilles tendinopathy via a longitudinal tendon-splitting approach resulted in good outcomes for patients after failure of initial conservative therapy. Recurrent calcification seems to be very common but shows no association with inferior outcomes or the return of symptoms.

**Level of Evidence:**

Level IV, retrospective case series.

## Introduction

Many studies have addressed the etiology of insertional Achilles tendinopathy (IAT), yet a specific reason for it could not be determined.^4,10,15,16,32,34^ By now, it is considered a multifactorial pathology. Earlier studies see associations with systemic diseases such as hypertension, obesity, and diabetes.^10,35^ Individual biomechanical properties such as malalignment of the foot, individual training errors, or lower extremity stiffness can also provoke the development of IAT.^9,17,22^ Pathophysiologic changes underlying IAT are still a major field of study. IAT is often referred to in the literature as “insertional Achilles tendinitis.” Although some studies have shown an inflammatory process to the tendon, we still lack clear results to indicate this pathology as inflammation. Therefore, this term is misleading, but terminology is often not consistent.^33,34^ This article uses the terminology of “Achilles tendinopathy” as suggested by Maffulli et al.^20^ IAT is thought to be a failed healing response to chronic overuse and mechanical overload of the tendon.^39^ High associations between IAT and a Haglund deformity are described, although it remains unclear if it is a factor that leads to IAT or if it is an adaptational process to it.^15^ The typical patient presents with tenderness, swelling, and pain at the tendon’s insertion to the calcaneus.^36^ The first approach should always be a nonoperative treatment, of which various methods are described. Conservative treatment options include eccentric training, extracorporal shockwave therapy, and platelet-rich plasma or corticosteriod injections.^5,8,13,21,29,37^ After 3 to 6 months of failed conservative treatment, surgery can be considered. The main aim is to debride the tendon, excise calcifications, and resect bony prominence to the calcaneus and reattach the tendon securely to its calcaneal footprint. Surgical techniques vary in their approach.^6,12,18,19,41^ A longitudinal tendon-splitting approach seems to be the most common among surgeons. The literature suggests superior outcomes for patients with double-row fixation over single-row constructs.^6^ Other studies failed to show differences in peak load to failure.^28^ Nevertheless, double-row constructs are known to provide a better restoration of the tendon insertion and ensure a bigger contact surface for the tendon to heal to bone.^31,36^ Not many studies have addressed the clinical outcome of patients undergoing a longitudinal tendon-splitting approach with double-row refixation after failed conservative treatment. The objectives of our study were to assess the (1) clinical outcome, including the American Orthopaedic Foot & Ankle Society (AOFAS) Hindfoot Score, Foot and Ankle Outcome Score (FAOS), and Foot Function Index (FFI); (2) pain relief using a visual analog scale (VAS) pain score; and (3) radiologic outcome with a special interest in recalcification after surgery; and to evaluate (4) the rate of recurrence and complications.

## Methods

This is a retrospective cohort study of prospectively collected data of patients who were operated on for IAT at our institution between 2013 and 2017. The study was approved by our institutional review board. In total, 49 patients could be identified who underwent surgery with double-row refixation using the Achilles SpeedBridge System (Arthrex, Naples, FL). All patients had confirmed IAT and did not respond to conservative therapy for more than 6 months. Diagnosis was based on the clinical presentation with accompanying radiographic findings and MRI scans. Patients were all older than 18 years of age. Exclusion criteria included any pathologic disorder to the lower limb, previous surgery on the foot of interest, neurologic disorders, and revision surgical procedures. We allowed a minimum follow-up of 18 months to allow full rehabilitation. Forty-two patients presented to the follow-up examination. Three patients refused participation and 4 patients remained unreachable due to wrong contact details or relocation. The mean age at time of surgery was 56.8 (range, 27-73) years. Twenty-six patients (62%) were female and 16 (38%) were male. The average follow-up time was 32.8 (range, 18-52) months. Patient demographics are described in Table 1. The indication for surgical treatment was confirmed IAT with failed conservative treatment for at least 6 months. The median time from onset of pain to the decision for surgical treatment was 24.0 (range, 9-180) months. For surgery, patients were placed in a prone position, giving the surgeon the best access to the Achilles tendon. A central tendon-splitting approach, first described by McGarvey et al,^23^ was used. After skin incision proximal to the Achilles tendon, the tendon was split longitudinal to access the insertion. Degenerative tendon tissue was excised. The tendon was not fully detached. It remained untouched on the very lateral and medial part of the insertion. None of our patients needed a flexor hallucis longus transfer due to excess debridement of the tendon of more than 50%. After excision of the Haglund exostosis and the bony spur with an osteotome, the tendon was reattached using a knotless double-row system with 4 bone anchors (SpeedBridge System). Postoperative management included a cast for 6 weeks with 2 weeks nonweightbearing in an equinus position, 2 weeks partial weightbearing, and 2 weeks full weightbearing in a plantigrade position, followed by a walker orthosis for another 2 weeks with full weightbearing. Data were collected using clinical examination and internationally validated scores. In our study, we included the VAS, AOFAS Hindfoot Score, FAOS, and FFI. All scores were evaluated before the surgery and again at the time of follow-up examination. We additionally performed an ankle radiograph to evaluate the postoperative result. Statistical analysis was performed using SPSS 25.0 (IBM Corp., Armonk, NY). Normal distribution of the data was confirmed by a Shapiro-Wilk test. Based on confirmation of normal distribution, statistical analysis was performed with either a paired Student *t* test or a Wilcoxon signed-rank test to analyze for significant differences in pre- to postoperative scores. *P* values less than .05 were considered to show statistical significance.

**Table 1.** Demographic Data.^a^

	Value
No. of patients	42
Male	16 (38.1%)
Female	26 (61.9%)
Age, y	56.8 ± 10.2 (27-73)
Follow-up, mo	32.8 ± 14.2 (18-52)
Median time from symptom onset to surgery, mo	24.0 ± 33.0 (9-180)

a Values are presented as mean ± SD (range) unless otherwise noted.

## Results

The scores of the VAS saw a significant decrease from 8.9 ± 1.0 (range, 7-10) preoperatively to 1.5 ± 1.4 (range, 0-9) postoperatively among patients (*P* < .01). Twenty-seven patients (64.3%) reached full pain relief (= VAS, 0). The AOFAS Hindfoot Score was 51.0 ± 12.5 (range, 30-73) preoperatively and 91.3 ± 14.3 (range, 46-100) at the time of follow-up (*P* < .01). For the FFI, we could see an improvement from 54.8 ± 15.5 (range, 24.0-88.8) to 8.1 ± 15.8 (range, 0-65.3) points (*P* < .01), as well as a significant improvement in every subscore (*P* < .05). All evaluated scores and results are listed in Table 2. Fifteen patients (35.7%) still suffered from pain at follow-up, 10 (23.8%) with moderate pain (= VAS, 1-3) and 5 (11.9%) with more severe pain (= VAS, >3). Three patients (7.1%) stated they did not feel any improvement of their symptoms at follow-up.

**Table 2.** Pre- and Postoperative Total and Subscores of the Collected Data.

Score	Preoperative	Postoperative	95% CI	*P* Value
VAS	8.9 ± 1.0	1.5 ± 2.5	6.4-8.4	<.01^a^
AOFAS	51.0 ± 12.5	91.3 ± 14.3	33.8-46.7	<.01^b^
Pain	6.5 ± 9.5	34.7 ± 9.0	23.2-33.3	<.01^a^
Function	35.9 ± 5.9	47.8 ± 4.9	8.6-14.4	<.01^b^
Alignment	9.9 ± 0.85	10.0 ± 0.0	—	>.05^a^
FFI	54.8 ± 15.5	8.1 ± 15.8	40.2-53.2	<.01^b^
Pain	73.3 ± 17.0	11.1 ± 21.2	53.2-71.1	<.01^a^
Disability	68.3 ± 22.1	9.0 ± 16.7	50.0-68.6	<.01^a^
Activity limitation	22.7 ± 18.8	4.1 ± 10.9	13.5-23.7	<.01^b^
FAOS				
Pain	36.5 ± 15.3	87.9 ± 18.1	43.5-59.3	<.01^b^
Other symptoms	60.4 ± 20.7	88.5 ± 15.1	21.4-34.7	<.01^b^
Quality of life	28.4 ± 14.9	74.5 ± 19.7	37.3-54.6	<.01^a^
Function, daily living	48.1 ± 18.4	91.5 ± 13.2	36.2-50.6	<.01^b^
Function, sports (n = 16)	31.6 ± 23.8	74.7 ± 32.9	27.6-58.6	<.01^a^

Abbreviations: AOFAS, American Orthopaedic Foot & Ankle Society Hindfoot Score; FAOS, Foot and Ankle Outcome Score; FFI, Foot Function Index; VAS, visual analog scale.

a Paired Student *t* test.

b Wilcoxon signed-rank test.

The postoperative radiograph showed recurrent calcifications in 30 patients (71.4%). We categorized calcifications based on their localization. Fourteen patients (33.3%) had singular proximal calcifications, 8 patients (19.0%) singular proximal with distal calcifications, 3 patients (7.1%) multiple proximal calcifications, and 5 patients (11.9%) multiple proximal with distal calcifications. Out of 30 patients with recurrent calcifications at follow-up, 24 (80.0%) rated their pain 0 on the VAS score, whereas 9 out of 12 patients without signs of new calcifications had a VAS score of greater than 0 (Table 3). There were no major complications among the patients who presented to our follow-up examination. Nevertheless, we identified 1 patient who did not present to our follow-up examination, who had a questionable irritation due to the implant where the implant had to be removed. Refixation was then achieved with bioresorbable components. Additionally, we saw 2 patients with superficial wound infection, who were treated conservatively with antibiotics. We also identified 1 patient who suffered from hypertrophic scar tissue and was therefore limited in shoe selection.

**Table 3.** Apportionment of Patients With and Without Persistent Pain and Their Follow-Up Radiograph Findings.^a^

New Radiograph Findings	Pain (VAS > 0)	No Pain (VAS = 0)
Yes: 30 (71.4)	6 (14.3)	24 (57.1)
No: 12 (28.6)	9 (21.4)	3 (7.1)

Abbreviations: VAS, visual analog scale.

a Values are presented as number (%).

## Discussion

Although the exact causes of IAT still remain unclear, multiple therapeutic approaches exist. This study aimed to investigate the outcome of patients who underwent surgical therapy through a longitudinal tendon-splitting approach with double-row refixation of their Achilles tendon. We were able to show that this treatment sees beneficial outcomes for patients regarding pain, disability, and their life quality. The findings of this study confirm the value of tendon debridement, spur, and Haglund removal with refixation of the tendon as operative treatment for patients with resistance to conservative therapy. Similar findings in earlier studies support these findings.^6,12,19,26^

Our AOFAS score (91.3) results were superior to those reported by Ettinger et al^6^ (86.5) on 40 patients and Johnson et al^12^ (89.0) on 22 patients. Looking at the study of Ettinger et al,^6^ 7 patients had double-row fixation of their tendon. They were able to show better outcomes among this collective compared with other fixation techniques. The results of patients with double-row fixation were even superior to ours (94.4). Rigby et al^30^ found a postoperative AOFAS score of 90 among 43 patients with an average follow-up of 24 months. Their preoperative VAS score was considerably lower with 6.8 points, compared with ours of 8.9. The postoperative score of 1.3 was comparable with 1.5 found in our study. Nunley et al^26^ reported an AOFAS score of 96.4% after 4 years and a 96% satisfactory rate among patients after a follow-up of 7 years.

Complications were reported in 11% of patients in a study with 432 patients by Paavola et al,^27^ with wound healing problems (3.2%) and superficial wound infection (2.5%) being the major complaints after surgery. This study included both insertional and noninsertional Achilles tendinopathy. A recent study by Hörterer et al^11^ looked at complications following midline incision to address Achilles tendon pathologies. They found a complication rate of 14% in 118 patients, the majority of which were surgical site infections (75%), followed by limitation in shoe selection (41%). Among our patients, we saw wound infection in 2 patients and hypertrophic scar tissue in 1 patient. As tendon calcification at the insertion site progresses after surgical therapy, this number is likely to increase with longer follow-up.

**Figure 1.**
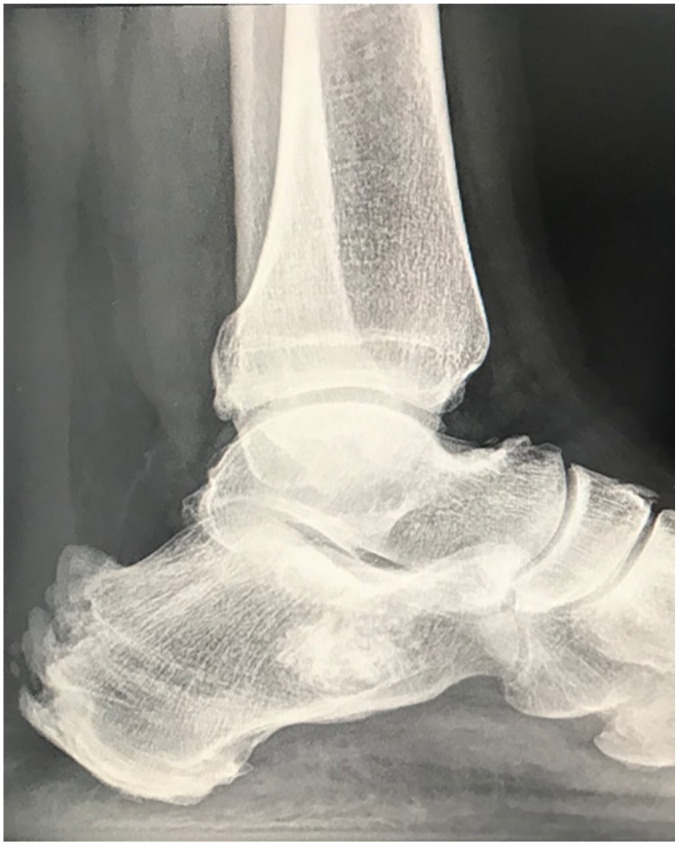
Sixty-two-year-old male 54 months postsurgery with full remission of symptoms but excessive new calcification to the Achilles tendon insertion at follow-up.

We found recurrent calcifications in 71.4% of patients. Nunley et al^26^ described new calcifications in 50% of patients. Interestingly, we could not identify any link between recurrent calcifications and the return or persistence of symptoms (Figure 1). Quite the contrary was the case. The majority of patients with persistent pain showed no new calcification at the insertion site of their tendon, and the majority of symptom-free patients showed signs of new calcification. As the surgery targets the pathologic calcified changes of the tendon, they are held responsible for the pain, but it seems that the clinical influence of these calcifications is yet to be determined. They might not be as relevant for the development of symptoms as it is believed. None of the studies to date have investigated if there is any link between the extent of calcification and severity of clinical symptoms. There is a possibility that calcifications are not the main factor for developing pain at the insertion site. Moreover, studies have shown that there is a reasonable amount of people who have asymptomatic calcification and spurs.^14,25^ Some believe that the pain derives from the neovascularization of the tendon from the paratenon and symptomatic tendinopathy is a result of ingrowth of new sensory and sympathic nerves accompanying these neovessels.^2,7^ Knobloch et al^16^ proofed significantly higher microcirculatory blood flow in pathologic Achilles tendons. Van Sterkenburg et al^40^ explain the favorable outcome of the operative treatment with the denervation of the tendon and paratenon. This would further explain the findings of asymptomatic new calcifications at follow-up in our study.

In our collective, we saw a difference in the expectation of patients on their postoperative results. The majority of patients in this study were aged 50 years and older, with only a few young patients. Only 11.9% of patients performed athletic activities before surgery. Patients with no athletic activities before surgery were able to meet their goal of postoperative activity by setting their level of low. Looking at physically more active patients, their priority is returning to their prior activity level. In our own experience, this goal was hard to achieve. Especially running is still painful for some patients, thus forcing them to switch to sports that are less demanding for the Achilles tendon, such as cycling or swimming. We do know that Achilles tendinopathies often occur in recreational and competitive athletes, but little is known about the specific outcome of this collective.

There is controversy regarding whether a double-row fixation is beneficial for patients. Studies on the biomechanical properties are not concordant and show different results.^1,28^ Early weightbearing with sooner start of rehabilitation might be one of the biggest benefits of this technique.^30^ Evidence in rotator cuff repairs does not show differences in functional outcomes, but big comparative studies are still missing.^3,24,38^

There are some limitations to our study that have to be addressed. First is a possible attrition bias, as it was not possible to examine all patients who underwent this surgical procedure. Moreover, a mean follow-up time of 32.8 months is rather short to adequately describe the full outcome of that therapy. Still little is known about the long-term outcome of patients, even if this and various other studies have already proofed the safety and efficiency of tendon debridement and refixation with a medium-term follow-up. We still lack sufficient data on longer follow-ups (>10 years). We do know that calcifications recur, so there is a high risk that symptoms also return. Therefore, the medium-term results of this study have to be interpretated with caution. Furthermore, measuring pain is always a challenging task to do, as it is a subjective parameter and therefore can result in big differences of how patients suffer from their clinical picture. Last, 42 patients is a small number to sufficiently assess the surgical outcome.

## Conclusion

Operative treatment for IAT using a longitudinal tendon-splitting approach shows good outcomes for patients who failed conservative therapy and should be considered for such cases. Recurrent calcification seems to be common but does not appear to be associated with inferior postoperative outcomes at medium-term follow-up.

## Supplemental Material

sj-pdf-1-fai-10.1177_10711007211002814 – Supplemental material for Clinical and Radiological Outcomes of Operative Therapy in Insertional Achilles Tendinopathy With Debridement and Double-Row Refixation Click here for additional data file. Supplemental material, sj-pdf-1-fai-10.1177_10711007211002814 for Clinical and Radiological Outcomes of Operative Therapy in Insertional Achilles Tendinopathy With Debridement and Double-Row Refixation by Fabian Greiner, Hans-Jörg Trnka, Michel Chraim, Elena Neunteufel and Peter Bock in Foot & Ankle International
